# Increased expression of the pluripotency markers sex-determining region Y-box 2 and Nanog homeobox in ovarian endometriosis

**DOI:** 10.1186/1477-7827-12-42

**Published:** 2014-05-18

**Authors:** Yong Song, Li Xiao, Jing Fu, Wei Huang, Qiushi Wang, Xianghui Zhang, Shiyuan Yang

**Affiliations:** 1 Department of Obstetrics and Gynecology, West China Second University Hospital of Sichuan University, Chengdu 610041, Sichuan, P. R. China

**Keywords:** Endometriosis, Sex-determining region Y-box 2, NANOG, Octamer-binding protein 4

## Abstract

**Background:**

The precise etiology of endometriosis is not fully understood; the involvement of stem cells theory is a new hypothesis. Related studies mainly focus on stemness-related genes, and pluripotency markers may play a role in the etiology of endometriosis. We aimed to analyze the transcription pluripotency factors sex-determining region Y-box 2 (SOX2), Nanog homeobox (NANOG), and octamer-binding protein 4 (OCT4) in the endometrium of reproductive-age women with and without ovarian endometriosis.

**Methods:**

We recruited 26 women with laparoscopy-diagnosed ovarian endometriosis (endometriosis group) and 16 disease-free women (control group) to the study. Endometrial and endometriotic samples were collected. SOX2, NANOG, and OCT4 expression were analyzed with quantitative real-time polymerase chain reaction, western blotting, and immunohistochemistry.

**Results:**

Compared to the control group, SOX2 mRNA and protein expression was significantly higher in the eutopic endometrium of participants in the endometriosis group. In the endometriosis group, SOX2 and NANOG mRNA and protein expression were significantly increased in ectopic endometrium compared with eutopic endometrium; there was a trend towards lower *OCT4* mRNA expression and higher OCT4 protein expression in ectopic endometrium.

**Conclusions:**

The transcription pluripotency factors SOX2 and NANOG were overexpression in ovarian endometriosis, their role in pathogenesis of endometriosis should be further studied.

## Background

Endometriosis is a chronic disease characterized by the presence of ectopic endometrial implants [1]. Current treatments such as medication or surgery are effective to an extent; however, some patients experience infertility, pain from ineffective treatment, recurrence, and even malignancy. Thus, identification of the mechanisms underlying the pathogenesis of endometriosis will facilitate the development of more effective treatment for this disorder.

Despite considerable investigation, the precise etiology and pathogenesis of endometriosis is unknown. To date, the leading theories are retrograde menstruation, coelomic metaplasia, embryonic cell rest, and lymphovascular metastasis [2]. However, none of them can annotate the pathogenesis to a point for all types of endometriosis. Current research involving stem cells should shed some light on the puzzling mechanisms of this disease [3-6]. However, data on stem or progenitor cell function in endometriosis are scarce due to the technical limitations to stem cell research. Current studies mainly focus on stemness-related genes; the pluripotency markers sex-determining region Y-box 2 (SOX2), Nanog homeobox (NANOG), and octamer-binding protein 4 (OCT4) are the most often studied [7-9]. *SOX2* is a member of the *SOX* (SRY-related high mobility group [HMG] box) gene family that encodes transcription factors with a single HMG DNA-binding domain [10]. *SOX* genes bind to the minor groove in DNA to control diverse developmental processes and play critical roles in cell fate determination, differentiation, and proliferation [11]. OCT4 is a member of the POU domain transcription factor family and plays a key role in the regulation of self-renewal and pluripotency in embryonic stem cells and primordial germ cells [12], while *NANOG* is a homeobox gene and has essential roles in maintaining self-renewal and the undifferentiated state of pluripotent stem cells [13]. In addition to maintaining the self-renewal ability of stem cells, SOX2, NANOG, and OCT4 are involved in cancer cell migration and invasion [13-15]. Aberrant SOX2, NANOG, and OCT4 expression has been demonstrated in endometriotic tissues, and OCT4 promoted endometrial cell migration activity [7-9]. However, these markers are not identified exclusively in women with ovarian endometriosis. Therefore, we aimed to examine and compare SOX2, NANOG, and OCT4 expression in endometrial or endometriotic tissues from women with and without ovarian endometriosis.

## Methods

### Study population

The Medical Research Review Board of West China Second University Hospital, Sichuan University (Sichuan, China) approved the study, and written informed consent was obtained from the human participants of the study. The participants were reproductive age women and had regular menstrual cycles. None had received hormonal treatment within the previous three months. Participants with adenomyosis, leiomyomas, endometrial hyperplasia, genital tumors, and acute pelvic inflammatory disease were excluded. From May 2012 to December 2012, we recruited 42 women who had undergone simultaneous laparoscopy and hysteroscopy to the study. Of these, 26 women with deep ovarian endometriosis (diameter of cyst is from 3 cm to 5 cm) were laparoscopically diagnosed and further confirmed by pathology (endometriosis group). The r-AFS score was used for disease stage (17 stage III, and 9 stage IV). The remaining 16 (age 24–32 years, mean 27 years) were controls who had undergone simultaneous laparoscopy and hysteroscopy for infertility which had no visible evidence of endometriosis or adhesions during surgery (Table 1).

**Table 1:** Characteristics of study population

**Characteristics**	**Endometriosis group (n = 26)**	**Non-endometriosis group (n = 16)**
Age (yrs)	27.94 ± 3.45 (22–35)	26.90 ± 2.47 (24–32)
Body mass index (kg/m^2^)	20.81 ± 2.94	19.14 ± 2.05
Infertility duration (yrs)	3.06 ± 1.71	3.10 ± 1.85
**r-AFS staging (n)**		
Stage III	17	0
Stage IV	9	0

Eutopic and ectopic endometrial tissues from ovarian endometrioma and from normal control endometrium were obtained during surgery. Of the 16 control specimens, 10 were used for quantitative real-time polymerase chain reaction (PCR) and the remaining six for western blot and immunohistochemistry analysis. Of the 26 endometriosis specimens, 13 were used for quantitative real-time PCR and the remaining 13 for western blot and immunohistochemistry analysis. Tissues were stored in a microfuge tube at -80°C for PCR and western blot analysis, or immediately fixed in 10% buffered formalin and paraffin-embedded for immunohistochemical and hematoxylin and eosin staining. All participants were determined to be in the proliferative phase of their menstrual cycle as assessed by the timing of their last menstrual period and histological dating [16].

### RNA isolation, cDNA synthesis, and real-time PCR

Total RNA was extracted using TRIzol according to the manufacturer’s protocol (Life Technologies Inc., Carlsbad, CA, USA); the quality and concentration of purified RNA were analyzed using a NanoVue Plus spectrophotometer (Health-careBio-ScienceAB, Uppsala, Sweden). The nucleotide to protein ratios (A260:A280) of all samples were within the acceptable boundaries of 1.8 and 2.1. First-strand complementary DNA (cDNA) synthesis was performed with a PrimeScript RT Reagent Kit (TaKaRa Biotechnology, Dalian, China) used according to the manufacturer’s protocol at 37°C for 30 min, followed by deactivation at 85°C for 8 s. PCR was performed using primers synthesized by Sangon Biotech (Shanghai, China) (Table 2). cDNA samples were diluted 10-fold for PCR. Each well of the PCR plate contained 5 μL EvaGreen Supermix (Bio-Rad Laboratories, Hercules, CA, USA), 1 μL of each primer (10 μmol/L), and 3 μL diluted cDNA. Amplification was performed over 39 cycles of 95°C for 30 s, 95°C for 5 s, and 60°C for 10 s; melting curve analysis from 65°C to 95°C at a rate of 5 s per step was performed to determine data quality using a CFX96 Real-Time PCR Detection System (Bio-Rad Laboratories). Only data with single melting peaks were included in the final analysis. All experiments were performed in triplicate. The threshold cycle values were normalized to the threshold value of human glyceraldehyde-3-phosphate dehydrogenase (*GADPH*) and the results expressed as the mean ± SD.

**Table 2:** Primer sequences used in quantitative real-time PCR

**Gene**	**Sense primer 5′ –3′**	**Antisense primer 5′ –3′**	**Genbank accession NM**
*GAPDH*	TGCACCACCAACTGCTTAGC	GGCATGGACTGTGGTGATGAG	NM_002046
*SOX2*	TACAGCATGTCCTACTCGCAG	GAGGAAGAGGTAACCACAGGG	NM_003106
*NANOG*	AAGGTCCCGGTCAAGAAACAG	CTTCTGCGTCACACCATTGC	NM_024865
*OCT4*	GCAGCGACTATGCACAACGA	CCAGAGTGGTGACGGAGACA	NM_002701

### Immunohistochemistry

Immunohistochemical staining was performed using monoclonal mouse anti-human SOX2 (ab75485, Abcam, Cambridge, MA, USA), polyclonal rabbit anti-human NANOG (ab80892, Abcam), and rabbit anti-human OCT4 (ab18976, Abcam) as primary antibodies. Briefly, serial sections were prepared, mounted on gelatin-coated slides, dried overnight at 37°C, deparaffinized in xylene, and rehydrated through a graded ethanol series. To retrieve the epitopes, the slides were immersed in citrate antigen retrieval buffer (pH 6) for 10 min at 120°C. After cooling, the sections were incubated with 3% H_2_O_2_ for 10 min to block endogenous peroxidase activity, blocked with 10% normal goat serum for 15–30 min, then incubated with the primary antibodies (1:100 SOX2, 1:400 NANOG, 1:100 OCT4) at 4°C overnight. Secondary biotinylated antibody and streptavidin-peroxidase conjugate were applied according to the manufacturer’s instructions (Beijing Zhongshan Biotech, Beijing, China); staining was visualized with diaminobenzidine, and then the sections were counterstained with hematoxylin and mounted. Colorectal carcinoma tissues were used as positive controls. Negative controls were performed by incubating sections with phosphate-buffered saline instead of primary antibodies.

### Western blot analysis

Total proteins were collected using radioimmunoprecipitation lysis buffer (P0013B, Beyotime Biotechnology, Shanghai, China) according to the manufacturer’s instructions. Protein concentration was determined using a bicinchoninic acid assay kit (Beyotime Biotechnology). Protein (30 ng) from each specimen underwent 10% sodium dodecyl sulfate–polyacrylamide gel electrophoresis and was transferred to polyvinylidene fluoride membranes (Millipore, Billerica, MA, USA). The membranes were blocked for 1 h in 5% defatted milk at room temperature. Subsequently, the membranes were incubated with monoclonal mouse anti-human SOX2 (1:200; ab75485, Abcam), polyclonal rabbit anti-human NANOG (1:300; ab80892, Abcam), OCT4 (1:500; ab18976, Abcam), and rabbit polyclonal anti-GAPDH (1:10000; bs-2188R, Bioss, Beijing, China) antibodies overnight at 4°C. Then, the membranes were incubated with horseradish peroxidase–conjugated secondary antibody for 1 h at room temperature. Proteins were detected with a chemiluminescence kit (Millipore) and the strength of the bands was analyzed with ImageJ2x (National Institutes of Health, Bethesda, MD, USA). Protein levels were normalized to that of the internal control GAPDH.

### Statistical analysis

Statistical analysis was performed using SPSS version 18.0 (SPSS, Chicago, IL, USA). All data are expressed as the mean ± SD. Differences between groups were evaluated with one-way analysis of variance with a post hoc test (Student–Newman–Keuls method); *P* < 0.05 was considered statistically significant (two-tailed).

## Results

### SOX2, NANOG, and OCT4 mRNA expression

**Figure 1:**
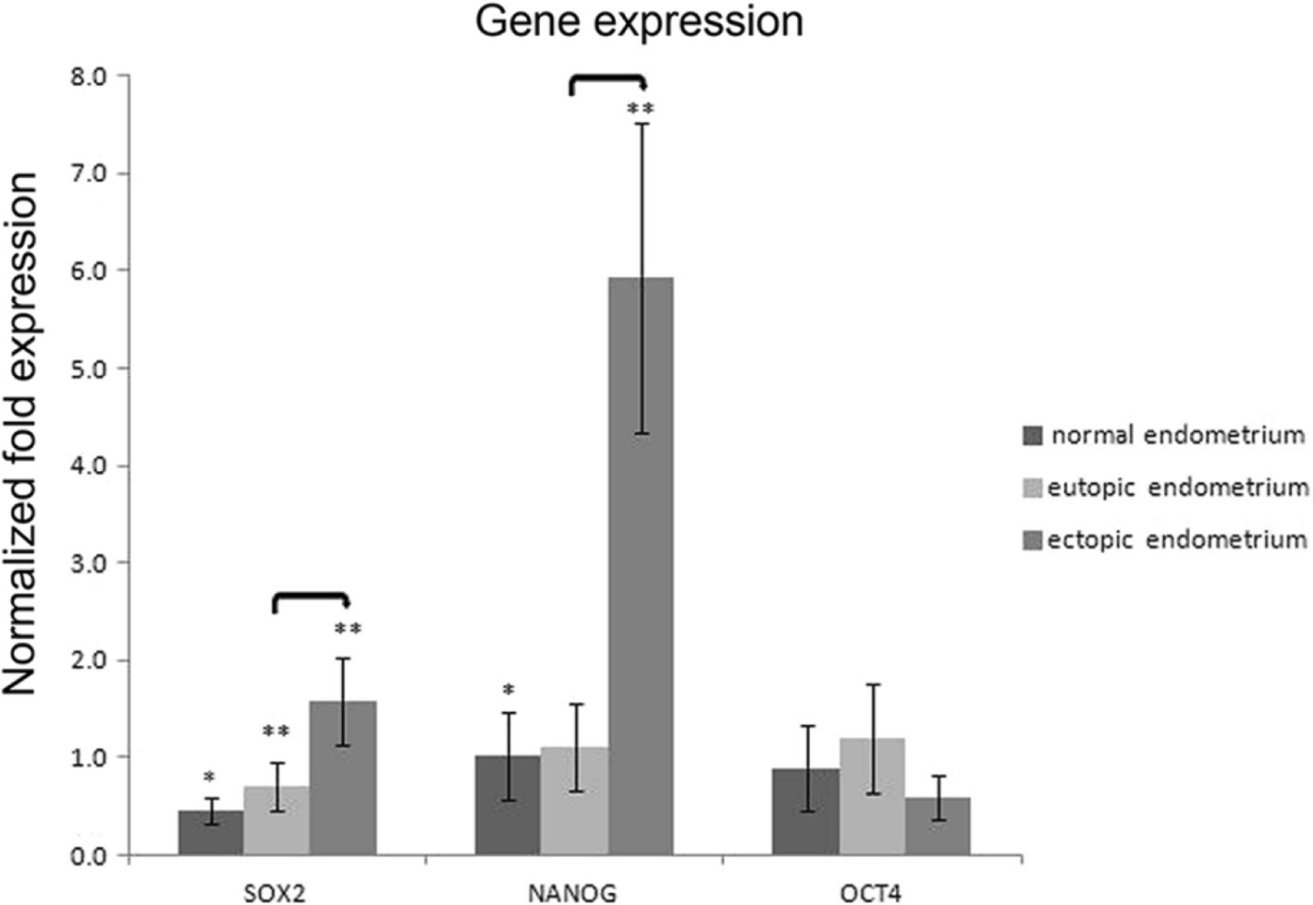
**Quantitative real-time PCR analysis of *SOX2*, *NANOG*, and *OCT4 *mRNA.** Analysis was carried out on normal endometrium (n = 10) and paired eutopic and ectopic endometrium specimens (n = 13). Double-asterisk values are significantly different from single-asterisk values. Asterisks denote significant differences between eutopic or ectopic endometrium and normal endometrium. Connecting lines denote significant differences between eutopic and ectopic endometrium. *P* < 0.05 was significant. Error bars denote SEM.

*SOX2* mRNA expression in the eutopic endometrium of ovarian endometriosis were significantly higher than that in normal endometrium (*P* = 0.02); expression *of NANOG* and *OCT4* mRNA in eutopic endometrium increased but no statistically significant when compared with normal controls. When compared to eutopic endometrium, *SOX2* and *NANOG* mRNA expression in ectopic endometrium was significantly increased (*P* = 0.004; *P* = 0.01, respectively), but *OCT4* mRNA expression in ectopic endometrium tended to be lower (*P* = 0.05). Both *SOX2* and *NANOG* mRNA expression in ectopic endometrium were significantly higher than that in normal endometrium (*P* = 0.001; *P* = 0.009, respectively), and only *OCT4* mRNA expression in ectopic endometrium tended to be lower (*P* > 0.05) (Figure 1).

### Expression and location of SOX2, NANOG, and OCT4 protein

**Figure 2:**
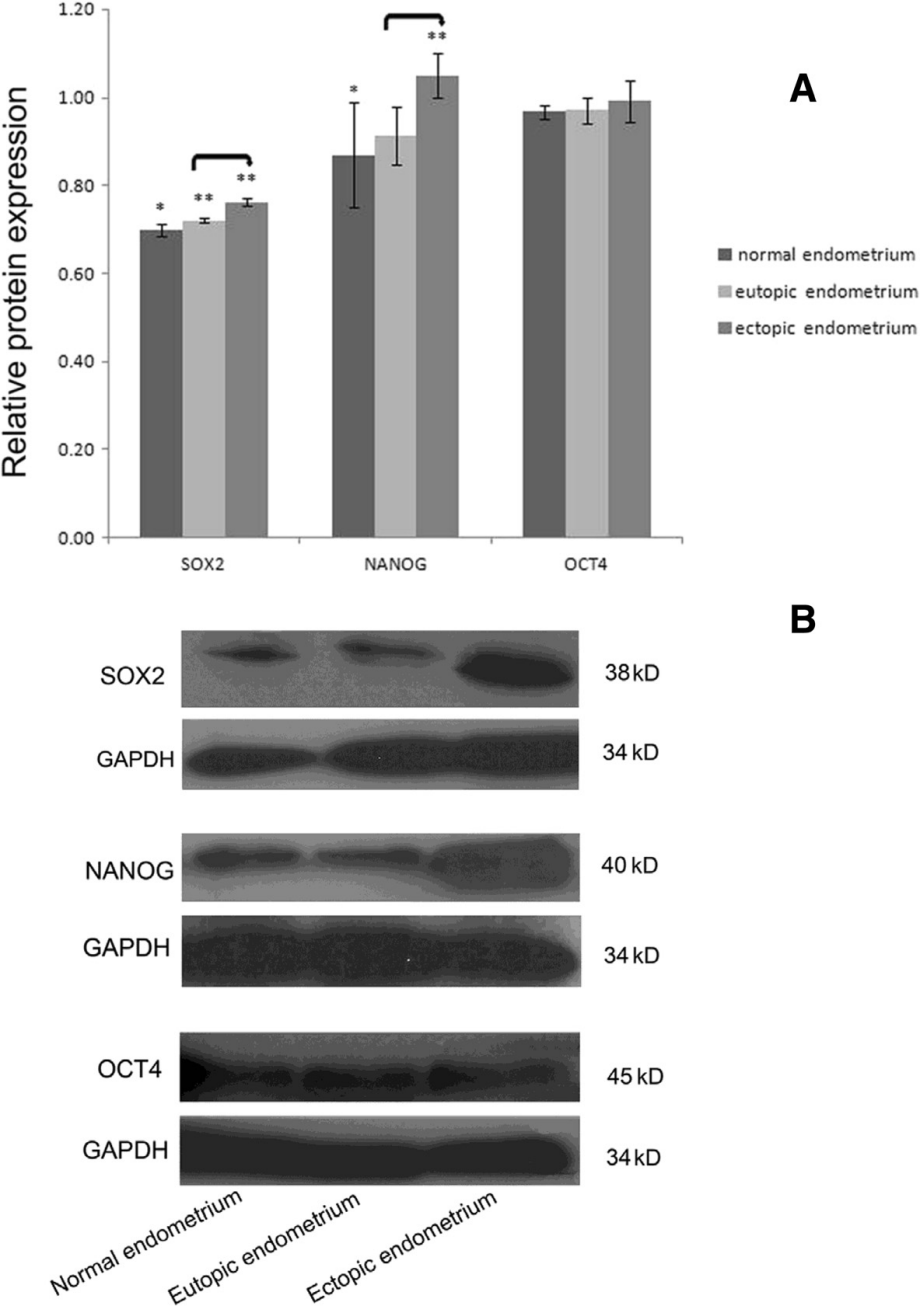
**Western blot analysis of SOX2, NANOG, and OCT protein. (A)** Relative levels of SOX2, NANOG, and OCT4 protein in normal endometrium (n = 6) and paired eutopic and ectopic endometrium (n = 13). Double-asterisk values are significantly different from single-asterisk values. Asterisks denote significant differences between eutopic or ectopic endometrium and normal endometrium. Connecting lines denote significant differences between eutopic and ectopic endometrium. *P* < 0.05 was significant. Error bars denote SEM. **(B)** Representative western blot of SOX2, NANOG, and OCT4 protein. GAPDH, Internal control.

Compared to normal endometrium, SOX2 protein expression in the eutopic and ectopic endometrium of ovarian endometriosis was significantly increased (*P* = 0.04; *P* = 0.009, respectively); NANOG protein expression in ectopic endometrium was statistically significantly different (*P* = 0.04); OCT4 protein expression in eutopic and ectopic endometrium tended to be higher (*P* > 0.05). Both SOX2 and NANOG protein expression in ectopic endometrium were significantly higher than that in eutopic endometrium (*P* = 0.01; *P* = 0.007, respectively), while only OCT4 protein expression in ectopic endometrium tended to be higher (*P* > 0.05) (Figure 2).

**Figure 3:**
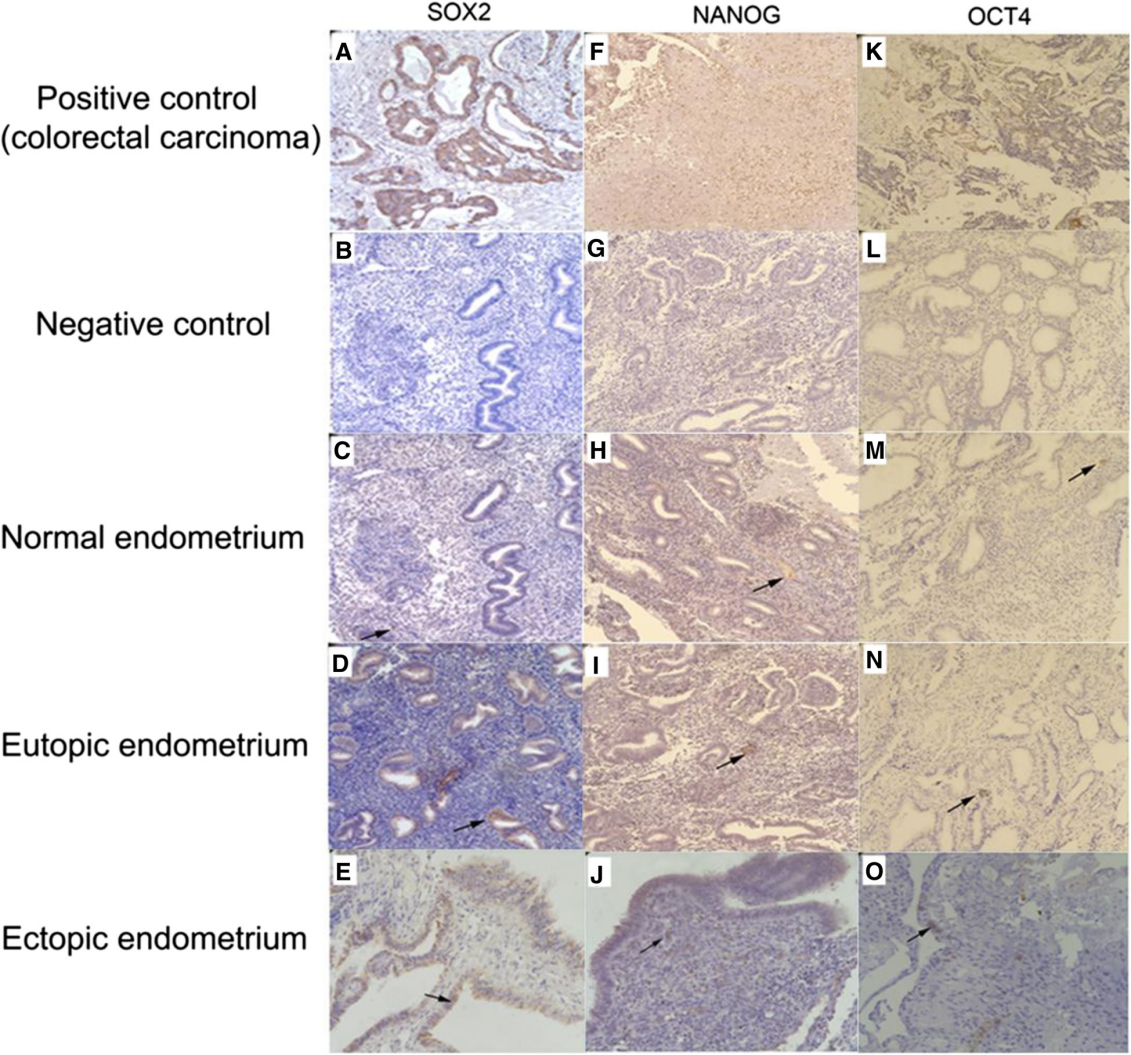
**SOX2, NANOG, and OCT4 immunohistochemical staining.** Paraffin-embedded normal (n = 6) and paired eutopic and ectopic endometrium specimens (n = 7) were examined. SOX2, NANOG, and OCT4 immunostaining in normal endometrium **(C, H, M)** of control and in eutopic endometrium **(D, I, N)** and ectopic endometrium **(E, J, O)** of endometriosis, and positive control **(A, F, K)** and negative control **(B, G, L)** were revealed, respectively (×400 magnification). Arrows denote positive staining.

SOX2, NANOG, and OCT4 protein were mainly expressed in the nuclei of glandular epithelial or stromal cells of ectopic and eutopic endometrium of ovarian endometriosis and normal control endometrium, with low expression detected in the cytoplasm of both cell types (Figure 3).

## Discussion

SOX2 and OCT4 are transcription factors essential for maintaining the self-renewal and pluripotent phenotype of embryonic stem cells [12,17]. Previous studies have revealed that they are also key factors in the reprogramming of somatic cells to a pluripotent state [18]. Similarly, NANOG can maintain the pluripotency and self-renewal characteristics of embryonic stem cells [19]. It has also been verified that these factors are involved in cancer cell migration and invasion [13,15,20]. Several other studies have also demonstrated that they are expressed in human normal endometrium, endometriotic tissues, and in endometrial cancer [7-9,21].

Emerging evidence suggests that there is decreased apoptosis, increased proliferation, and higher migration and invasion ability in the eutopic endometrium of endometriosis [22,23]. The aberrant SOX2 expression in ovarian endometriosis in our study, consistent with that reported previously [7,8], may indicate a stem cell origin of endometriosis. As a pluripotent transcription factor, SOX2 overexpression in eutopic and ectopic endometrial specimens of ovarian endometriosis was expected to result in increased proliferation and inhibit apoptosis by targeting apoptosis-related proteins as it does in neural stem cells and prostate cancer [24,25]. As SOX2 affected migration/invasion by downregulating matrix metalloproteinase-2 in colorectal cancer [15], we believe that its overexpression in ovarian endometriosis may be a potential mechanism that leads to the intrinsic differentiation of eutopic endometrial cells from that in normal endometria, and play an important role in the disease pathogenesis.

In this study, NANOG expression was significantly increased in the ectopic endometrium of women with ovarian endometriosis. This result differs from that of previous studies [7,9]. The reason may be related to the study population, the ages of the participants, or their menstrual cycles.

Endometriosis is usually considered an estrogen-dependent disorder [1]. In ectopic endometrium, estradiol (E2) is overproduced by aromatase and is not metabolized because the ectopic endometrium lacks 17β-hydroxysteroid dehydrogenase type 2 activity. Two recent studies support the positive link between high levels of E2 and SOX2 or NANOG expression [26,27]. SOX2 and NANOG overexpression may foster self-renewal and increase cell survival in ovarian endometrial tissues that can further facilitate ectopic tissue growth. More importantly, data from large cohort studies indicate that endometriosis patients have an increased risk of ovarian cancer [28,29]. Recently, two studies revealed that SOX2 overexpression and advanced ovarian cancer were closely associated [30,31]. Another study demonstrated that NANOG was involved in ovarian tumorigenesis through migration and invasion [13]. In this study, NANOG expression in ectopic endometrium was nearly six times that in eutopic endometrium, and SOX2 expression was four times in ectopic endometrium. The results indicate that NANOG alone or in combination with SOX2 may contribute to the transformation of ovarian endometriosis to ovarian cancer.

In our study, OCT4 expression was similar among normal endometrium and eutopic and ectopic endometrium of endometriosis. It has been established that OCT4 is a key transcription factor in the regulation of self-renewal and pluripotency in embryonic stem cells and in the reprogramming of somatic cells to a pluripotent state. Chang *et al*. found that OCT4 was upregulated in human ectopic endometriotic tissues and that it contributed to ectopic endometrial growth by stimulating endometrial cell migration activity [9]. However, we could not verify this in the present study, which might have been due to the study population and the menstrual cycles of the participants; therefore, further studies would require a larger sample size.

In our study, there were some drawback like sample size is not large and amount of endometrial tissue cannot match all experimental items that result in some index not reach statistical significance. Infertile women are chosen as control is not perfect, however, it’s hard to get endometrial tissue from normal fertile women that undergo laparoscopy and hysteroscopy procedure. A larger sample size and the functional research of SOX2 and NANOG in pathogenesis of endometriosis is anticipated in our further study.

## Conclusions

In reproductive-age women with ovarian endometriosis, the transcriptional factor SOX2 and NANOG are over expression. Future studies is need to determine their role in pathogenesis of ovarian endometriosis.

## Abbreviations

E2: Estradiol; MMP: Matrix metalloproteinases; OCT4: Octamer-binding protein 4; SOX2: Sex-determining region Y-box 2.

## Competing interests

The authors declare that they have no competing interests.

## Authors’ contributions

YS designed the study, performed experiments and drafted the manuscript. LX performed experiments, did statistic analysis and drafted the manuscript. JF and QW designed the study and performed experiments. WH designed the study and helped to draft the manuscript. XZ and SY collected the tissue samples and participated in the study. All authors read and approved the final manuscript.
